# Niche modeling reveals life history shifts in birds at La Brea over the last twenty millennia

**DOI:** 10.1371/journal.pone.0227361

**Published:** 2020-01-16

**Authors:** Robert M. Zink, Sebastian Botero-Cañola, Helen Martinez, Katelyn M. Herzberg

**Affiliations:** 1 School of Natural Resources, University of Nebraska, Lincoln, Nebraska, United States of America; 2 School of Biological Sciences, University of Nebraska, Lincoln, Nebraska, United States of America; 3 Nebraska State Museum, University of Nebraska, Lincoln, Nebraska, United States of America; 4 Harold W. Manter Laboratory of Parasitology, University of Nebraska, Lincoln, Nebraska, United States of America; 5 College of Agriculture and Natural Resources, University of Nebraska, Lincoln, Nebraska, United States of America; Universitat Trier, GERMANY

## Abstract

A species presence at a particular site can change over time, resulting in temporally dynamic species pools. Ecological niche models provide estimates of species presence at different time intervals. The avifauna of La Brea includes approximately 120 species dating to approximately 15,000 years ago. Niche models predicted presence at the Last Glacial Maximum for over 90% of 89 landbird species. This confirms that niche modeling produces sensible range estimates at the Last Glacial Maximum. For 97 currently local species that are as yet undocumented at La Brea over 90% were predicted to occur; absence is due to insufficient study, lack of the ecological niche, transient occurrence or a behavioral ability to avoid entrapment. Our 366 niche models provide a prospective checklist of the landbird fauna of La Brea. The models indicate fluidity in life history strategies and a higher proportion of resident birds at the LGM (88% to 60%). We evaluated a subset of 103 species in breeding and winter periods using two climate models (MIROC−ESM, CCSM4) with a variety of differing parameters, finding differences in 5% of the niche models. Niche breadths in bark-foraging birds changed little between the present and LGM, suggesting that greater species diversity at the LGM was due to greater niche availability rather than contractions of niche breadths (i.e., niche partitioning).

## Introduction

Lists of species from specific localities form the basis for many ecological analyses such as characterizing geographic patterns in species diversity and identifying high-priority conservation areas. Species lists from modern and recent localities have also been used to estimate species turnover over time [1,2]. Niche modeling is a major research tool for predicting past and future species’ distributions [3,4], which aid in understanding how community species composition changes over time. In most niche-model studies, predicted species distributions in the past were not informed by actual locality information, but rather through identifying where the current niche conditions existed at that earlier time. Some recent studies have attempted to integrate paleodata, such as historical localities obtained from microfossils, to improve model predictions in past environments [5–8]. However, paleo records are lacking for many species, and most studies rely on projected ranges without direct testing of their accuracy. To validate such approaches, a densely sampled archaeological or Holocene site with a verified list of species, which can be compared to projected distributions from niche modeling using current records across a large sample of species, is needed.

Identified skeletal remains of animals entombed in the sticky tar at La Brea provide an opportunity to test whether distributions predicted by ecological niche models at the Last Glacial Maximum (LGM) overlap the site. The fauna of La Brea is a heterogeneous assemblage and not likely a random sample of what was in the area at a given time. For example, mammals are over-represented by large predators, such as dire wolves (*Canus dirus*) and sabre-tooth cats (*Smilodon fatalis*), which came to prey on entrapped ungulates, including camels (*Camelops hesternus)* and mastodons (*Mammut americanum)*. In fact, over 90% of identified entombed large mammals are carnivores, leading La Brea to be known as a “carnivore trap”. Whether this reflects the species community surrounding La Brea, or the allure of large carnivores to paleontologists, is unclear. Nonetheless, many vertebrates have been preserved during the period of 10 to 30 thousand years ago (ka).

The avian assemblage of La Brea (Table 1) was documented by Howard [9–11]; see http://www.tarpits.org/research-collections/collections/bird-collections. The list is scheduled to be updated (K. Campbell, in litt. 22 Sept. 2017). Miller [12] noted that identified avian remains are biased towards large-bodied, mostly raptorial taxa. A total of 122 species (including 21 extinct) were identified; in five cases, tentative identifications were made to the species level owing to the difficulty in identifying many species, especially passerines, from skeletons alone. Several other specimens were identified to genus only. For example, no New World Wood Warblers were identified to species, instead warbler skeletons were referred to simply as “Indeterminate Parulinae”. The same generic identification was offered for orioles (“*Icterus* spp.”) and some sparrows (“Indeterminate Fringillidae”). In some cases, it appears that geography played a role. For example, the list includes the red-shafted flicker (*Colaptes auratus cafer*), which likely cannot be told apart from the yellow-shafted flicker (*Colaptes a*. *auratus*) by skeletal features alone (pers. obs.); however, only the former (sub)species occurs in that part of California, and therefore it is likely that the subspecies identification was based on current ranges. The list of species represents the pooling of individuals from different pits, which themselves differ in age and extent [11]; K. Campbell (in litt.) noted that the fossils from Rancho La Brea range from 10000 to 40000 yrs.

**Table 1:** List of species analyzed, presence in La Brea deposits, number of specimens and tar pit layers for each species, distance in km from La Brea to nearest predicted breeding occurrence at Last Glacial Maximum, distance in km from La Brea to nearest predicted wintering occurrence at Last Glacial Maximum, residency status at La Brea at the Last Glacial Maximum, and present status at La Brea (within 100 km). An asterisk indicates that the condition shown is most probable given the data.

Common_name	Scientific name	Present at La Brea	Number of specimens, number of pits	LGM: Distance (km) to predicted breeding	LGM: Distance (km) to winter prediction	LGM status	Present status
Acorn Woodpecker	*Melanerpes formicivorus*	N	NA	0	0	resident	resident
Allen’s Hummingbird	*Selasphorus sasin*	N	NA	0	0	resident	resident
American Crow	*Corvus brachyrhynchos*	Y	22,7	5	0	resident	resident
American dipper	*Cinclus mexicanus*	N	NA	5	0	resident	resident
American Goldfinch	*Spinus tristis*	Y	3,1	0	0	resident	winter
American Kestrel	*Falco sparverius*	Y	79,11	0	0	resident	resident
American Pipit	*Anthus rubescens*	N	NA	0	0	resident	winter
American Robin	*Turdus migratorius*	Y	18,3	0	0	resident	resident
Anna’s Hummingbird	*Calypte anna*	N	NA	0	0	resident	resident
Ash-throated flycatcher	*Tyrannus vociferans*	Y	4,1	0	0	resident	breeding
Bald Eagle	*Haliaeetus leucocephalus*	Y	175,10	5	0	resident	winter
Band-tailed Pigeon	*Patagioenas fasciata*	Y	3,3	0	0	resident	resident
Barn Owl	*Tyto alba*	Y	205,10	0	0	resident	resident
Barn Swallow	*Hirundo rustica*	N	NA	0	0	resident	breeding
Bell’s Sparrow	*Artemisiospiza belli*	Y	6?	0	0	resident	resident
Bell’s Vireo	*Vireo bellii*	N	NA	0	0	resident	breeding
Belted Kingfisher	*Megaceryle alcyon*	N	NA	0	0	resident	winter
bendire’s thrasher	*Toxostoma bendirei*	N	NA	80	200	breeding	not present
Bewick’s Wren	*Thryomanes bewickii*	N	NA	0	0	resident	resident
Black Phoebe	*Sayornis nigricans*	N	NA	0	0	resident	resident
Black Swift	*Cypseloides niger*	N	NA	0	15	resident	breeding
Black-backed woodpecker	*Picoides arcticus*	N	NA	25	50	resident	not present
Black-chinned Hummingbird	*Archilochus alexandri*	N	NA	0	0	resident	breeding
Black-chinned Sparrow	*Spizella atrogularis*	N	NA	0	150	breeding	breeding
Black-headed Grosbeak	*Pheucticus melanocephalus*	Y	1,1	0	0	resident	breeding
Black-throated Gray Warbler	*Setophaga nigrescens*	?	?	>500	700	not present	breeding
Black-throated Sparrow	*Amphispiza bilineata*	Y	4,1	45	80	resident	breeding*
Blue Grosbeak	*Passerina caerulea*	N	NA	70	0	resident	breeding
Blue-gray Gnatcatcher	*Polioptila caerulea*	N	NA	0	0	resident	resident
Bohemian Waxwing	*Bombycilla garrulus*	N	NA	100	50	winter	winter
Brea owl	*Oraristrix brea*	Y	23,1			resident?	not present (extinct)
Brewer’s Blackbird	*Euphagus cyanocephalus*	?	?	0	0	resident	resident
Brown Creeper	*Certhia americana*	N	NA	0	0	resident	resident*
Brown-headed cowbird	*Molothrus ater*	Y	1,1	0	0	resident	resident
Bullock’s Oriole	*Icterus bullockii*	?	?	0	0	resident	breeding
Burrowing Owl	*Athene cunicularia*	Y	228,9	50	0	resident	resident
Bushtit	*Psaltriparus minimus*	N	NA	0	0	resident	resident
Cactus Wren	*Campylorhynchus brunneicapillus*	N	NA	500	20	winter	resident
California Condor	*Gymnogyps californianus*	N	NA	0	0	resident	breeding
California Gnatcatcher	*Polioptila californica*	N	NA	0	0	resident	resident
California Quail	*Callipepla californica*	Y	138,7	0	0	resident	resident
California Scrub-Jay	*Aphelocoma californica*	Y	8,3	0	0	resident	resident
California Thrasher	*Toxostoma redivivum*	Y	6,3	0	0	resident	resident
California Towhee	*Melozone crissalis*	Y	2,1	0	0	resident	resident
Calliope Hummingbird	*Selasphorus calliope*	N	NA	25	0	resident	breeding
Canyon Wren	*Catherpes mexicanus*	N	NA	0	0	resident	resident
Cassin’s Finch	*Haemorhous cassinii*	N	NA	15	20	resident	resident
cassin’s kingbird	*Tyrannus vociferans*	N	NA	0	20	resident	breeding
Cassin’s Vireo	*Vireo cassinii*	N	NA	0	5	resident	breeding
Cedar Waxwing	*Bombycilla cedrorum*	Y	?	5	0	resident	winter
Chestnut-backed Chickadee	*Poecile rufescens*	?	?	0	0	resident	not present
Chihuahuan Raven	*Corvus cryptoleucus*	Y	1,1	200	500	not present	not present
Chipping Sparrow	*Spizella passerina*	Y	6,6	0	0	resident	resident
Clark’s nutcracker	*Nucifraga columbiana*	Y	2,1	35	20	resident	resident*
Cliff Swallow	*Petrochelidon pyrrhonota*	N	NA	0	0	resident	breeding
Common Poorwill	*Phalaenoptilus nuttallii*	Y	7,1	0	0	resident	resident
Common Raven	*Corvus corax*	Y	114,13	0	0	resident	resident
Common Yellowthroat	*Geothlypis trichas*	?	?	30	0	resident	resident
Cooper’s Hawk	*Accipiter cooperii*	Y	52,8	0	0	resident	resident
Costa’s Hummingbird	*Calypte costae*	N	NA	0	0	resident	resident
Crissal Thrasher	*Toxostoma crissale*	N	NA	150	100	not present	not present
Dark-eyed Junco (Oregon)	*Junco hyemalis*	N	NA	0	0	resident	resident
Downy Woodpecker	*Picoides pubescens*	N	NA	0	0	resident	resident
Dusky Flycatcher	*Empidonax oberholseri*	N	NA	20	NC	breeding	breeding
Evening Grosbeak	*Coccothraustes vespertinus*	Y	1,1	5	0	resident	winter
Extinct blackbird	*Euphagus magnirostris*	Y	1,1	0	0?	resident?	not present (extinct)
Extinct Icterid	*Pandanaris convexa*	Y	1,1	0	0?	resident?	not present (extinct)
Extinct towhee	*Melozone angelensis*	Y	11,1	0	0?	resident?	not present (extinct)
Ferruginous Hawk	*Buteo regalis*	Y	127,13	100	0	resident	winter
Flammulated Owl	*Psiloscops flammeolus*	N	NA	15	0	resident	breeding
Fox Sparrow	*Passerella iliaca*	Y	2,1	0	0	resident	resident
Gambel’s Quail	*Callipepla gambelii*	N	NA	50	100	breeding	resident
Golden Eagle	*Aquila chrysaetos*	Y	960,12	0	0	resident	resident
Golden-crowned Kinglet	*Regulus satrapa*	N	NA	0	0	resident	winter
Golden-crowned Sparrow	*Zonotrichia atricapilla*	N	NA	0	0	resident	winter
Grasshopper Sparrow	*Ammodramus savannarum*	N	NA	10	0	resident	breeding
Gray Flycatcher	*Empidonax wrightii*	N	NA	20	NC	breeding	breeding
Great Horned Owl	*Bubo virginianus*	Y	128,12	2	0	resident	resident
Greater Roadrunner	*Geococcyx californianus*	Y	25,6	10	0	resident	resident
greater sage grouse	*Centrocercus urophasianus*	N	NA	55	80	breeding	not present
Green-tailed Towhee	*Pipilo chlorurus*	N	NA	15	0	resident	resident
Hairy Woodpecker	*Leuconotopicus villosus*	N	NA	0	0	resident	resident
Hammond’s Flycatcher	*Empidonax hammondii*	N	NA	0	NC	breeding	breeding
Hermit Thrush	*Catharus guttatus*	N	NA	15	0	resident	winter
Hermit Warbler	*Setophaga occidentalis*	?	?	0	10	resident	breeding*
Hooded Oriole	*Icterus cucullatus*	?	?	0	>500	breeding	breeding
Horned Lark	*Eremophila alpestris*	Y	1,1	10	0	resident	resident
House Finch	*Haemorhous mexicanus*	N	NA	0	150	resident	resident
House Wren	*Troglodytes aedon*	N	NA	0	0	resident	resident
Hutton’s Vireo	*Vireo huttoni*	N	NA	0	0	resident	resident
Lark Sparrow	*Chondestes grammacus*	Y	3,1	0	0	resident	resident
Lawrence’s Goldfinch	*Spinus lawrencei*	N	NA	0	0	resident	resident
Lazuli Bunting	*Passerina amoena*	N	NA	0	5	resident	breeding
LeConte’s Thrasher	*Toxostoma lecontei*	N	NA	100	100	not present	resident
Lesser Goldfinch	*Spinus psaltria*	N	NA	0	0	resident	resident
Lesser Nighthawk	*Chordeiles acutipennis*	N	NA	60	700	breeding	breeding
Lewis’s Woodpecker	*Melanerpes lewis*	Y	7,3	3	0	resident	winter
Lincoln’s Sparrow	*Melospiza lincolnii*	N	NA	20	0	resident	winter
Loggerhead Shrike	*Lanius ludovicianus*	Y	3,2	0	0	resident	resident
Long-eared owl	*Asio otus*	N	NA	0	0	resident	resident
MacGillivray’s Warbler	*Geothlypis tolmiei*	?	?	5	500	breeding	breeding
Marsh Wren	*Cistothorus palustris*	N	NA	0	0	resident	winter
Merlin	*Falco columbarius*	Y	16,8	25	0	resident	winter
Mountain Bluebird	*Sialia currucoides*	?	?	30	0	resident	resident*
Mountain Chickadee	*Poecile gambeli*	?	?	10	0	resident	resident
Mountain Quail	*Oreortyx pictus*	N	NA	0	0	resident	resident
Mourning Dove	*Zenaida macroura*	Y	30,6	0	0	resident	resident
Nashville Warbler	*Oreothlypis ruficapilla*	?	?	20	0	resident	breeding*
Northern Goshawk	*Accipiter gentilis*	Y	2,1	10	5	resident	winter
Northern Harrier	*Circus hudsonius*	Y	164,11	0	0	resident	resident
Northern Mockingbird	*Mimus polyglottos*	N	NA	35	0	resident	resident
Northern Pygmy-Owl	*Glaucidium gnoma*	Y	5,1	0	0	resident	resident
Northern Rough-winged Swallow	*Stelgidopteryx serripennis*	N	NA	0	0	resident	breeding
Northern Saw-whet Owl	*Aegolius acadicus*	Y	1,1	0	0	resident	resident
Nuttall’s Woodpecker	*Picoides nuttallii*	N	NA	0	0	resident	resident
Oak Titmouse	*Baeolophus inornatus*	N	NA	0	0	resident	resident
olive-sided flycatcher	*Contopus cooperi*	N	NA	0	0?	resident	breeding
Orange-crowned Warbler	*Oreothlypis celata*	?	?	0	0	resident	resident
Pacific Wren	*Troglodytes pacificus*	N	NA	0	0	resident	not present
Pacific-slope Flycatcher	*Empidonax difficilis*	N	NA	0	NC	breeding	breeding
Passenger Pigeon	*Ectopistes migratorius*	Y	3,3	125	NC	resident	not present (extinct)
Peregrine Falcon	*Falco peregrinus*	Y	29,9	0	0	resident	resident
Phainopepla	*Phainopepla nitens*	N	NA	0	0	resident	breeding
Pileated Woodpecker	*Dryocopus pileatus*	Y	1,1	0	0	resident	not present
Pine Siskin	*Spinus pinus*	Y	?	0	0	resident	resident
Prairie Falcon	*Falco mexicanus*	Y	24,10	40	0	resident	resident
Purple Finch	*Haemorhous purpureus*	N	NA	1200	0	winter	resident
Pygmy Nuthatch	*Sitta pygmaea*	N	NA	0	0	resident	resident
Red crossbill	*Loxia curvirostra*	N	NA	0	0	resident	winter
Red-breasted Nuthatch	*Sitta canadensis*	N	NA	0	0	resident	resident
Red-breasted Sapsucker	*Sphyrapicus ruber*	?	1,1?	0	0	resident	resident
red-shafted flicker	*Colaptes auratus*	Y	18,4	0	0	resident	resident
Red-shouldered Hawk	*Buteo lineatus*	?	?	0	0	resident	resident
Red-tailed Hawk	*Buteo jamaicensis*	Y	108,13	0	0	resident	resident
Red-winged Blackbird	*Agelaius phoeniceus*	?	?	0	0	resident	resident
Rock Wren	*Salpinctes obsoletus*	N	NA	20	0	resident	resident
Rough-legged Hawk	*Buteo lagopus*	Y	7,4	200	10	winter	not present
Ruby-crowned Kinglet	*Regulus calendula*	N	NA	0	0	resident	resident
rufous hummingbird	*Selasphorus rufus*	N	NA	0	0	resident	migrant
Rufous-crowned Sparrow	*Aimophila ruficeps*	N	NA	0	0	resident	resident
Sage Thrasher	*Oreoscoptes montanus*	Y	1,1	70	0	resident	not present
Savannah Sparrow	*Passerculus sandwichensis*	N	NA	0	0	resident	resident
Say’s Phoebe	*Sayornis saya*	N	NA	45	0	resident	resident
Scott’s Oriole	*Icterus parisorum*	N	NA	50	0	resident	winter
Sharp-shinned Hawk	*Accipiter striatus velox*	Y	5,4	5	0	resident	winter
Short-eared Owl	*Asio flammeus*	Y	157,12	40	0	resident	winter
Song Sparrow	*Melospiza melodia*	Y	10,1	0	0	resident	resident
sooty grouse	*Dendragapus fuliginosus*	N	NA	5	20	resident	not present
Spotted Owl	*Strix occidentalis*	N	NA	0	0	resident	resident
Spotted Towhee	*Pipilo maculatus*	Y	4,1	0	0	resident	resident
Steller’s Jay	*Cyanocitta stelleri*	Y	4,3	0	0	resident	resident
Summer Tanager	*Piranga rubra*	?	?	65	0	resident	not present
Swainson’s Hawk	*Buteo swainsoni*	Y	130,11	25	700	breeding	not present
Swainson’s Thrush	*Catharus ustulatus*	N	NA	0	1000	breeding	breeding
Townsend’s Solitaire	*Myadestes townsendi*	N	NA	15	0	resident	resident
Townsend’s Warbler	*Setophaga townsendi*	?	?	20	0	resident	winter
tree swallow	*Tachycineta bicolor*	N	NA	5	0	resident	resident
Tricolored Blackbird	*Agelaius tricolor*	?	?	0	0	resident?	resident
Turkey Vulture	*Cathartes aura*	Y	34,13	10	0	resident	resident
Varied Thrush	*Ixoreus naevius*	N	NA	0	0	resident	winter
Vaux Swift	*Chaetura vauxi*	N	NA	0	0	resident	migrant
Verdin	*Auriparus flaviceps*	N	NA	60	150	breeding	resident
Vesper Sparrow	*Pooecetes gramineus*	Y	1,1	75	0	resident	winter
Violet-green Swallow	*Tachycineta thalassina*	N	NA	0	0	resident	breeding
Warbling Vireo	*Vireo gilvus*	N	NA	250	0	winter	breeding
Western Bluebird	*Sialia mexicana*	?	7,2?	0	0	resident	resident
Western Kingbird	*Tyrannus verticalis*	N	NA	0	0	resident	breeding
Western Meadowlark	*Sturnella neglecta*	Y	125,11	10	0	resident	resident
Western Screech-Owl	*Megascops kennicottii*	Y	16,7	0	0	resident	resident
Western Tanager	*Piranga ludoviciana*	N	NA	0	0	resident	breeding
Western Wood-Pewee	*Contopus sordidulus*	N	NA	0	0	resident	breeding
White-breasted Nuthatch	*Sitta carolinensis*	N	NA	5	0	resident	resident
White-crowned Sparrow	*Zonotrichia leucophrys*	Y	6,1	0	0	resident	resident
white-headed woodpecker	*Picoides albolarvatus*	N	NA	20	0	resident	resident
White-tailed Kite	*Elanus leucurus*	Y	3,3	0	0	resident	resident
White-throated Sparrow	*Zonotrichia albicollis*	N	NA	500	0	winter	winter
White-throated Swift	*Aeronautes saxatalis*	N	NA	0	0	resident	resident
Wild Turkey	*Meleagris gallopavo*†	Y	599,12	30	0	resident	not present^2^
Williamson’s Sapsucker	*Sphyrapicus thyroideus*	?	?	20	0	resident	resident
Willow Flycatcher	*Empidonax traillii*	N	NA	0	NC	breeding	breeding
Wilson’s Warbler	*Cardellina pusilla*	?	?	30	1000	breeding	breeding
Wrentit	*Chamaea fasciata*	N	NA	0	0	resident	resident
Yellow-billed Cuckoo	*Coccyzus americanus*	N	NA	60	0	resident	not present
Yellow-billed Magpie	*Pica nuttalli*	Y	174,9	0	0	resident	not present
Yellow-breasted Chat	*Icteria virens*	N	NA	5	0	resident	breeding
Yellow-headed Blackbird	*Xanthocephalus*?	?	?	60	0	resident	resident
Yellow-rumped Warbler	*Setophaga coronata*	?	?	3	0	resident	resident*

What is puzzling about the La Brea record is the absence of many passerine and other small-bodied birds that are today common in western North America, including hummingbirds, tanagers, nuthatches, titmice, vireos, wrens, thrushes, swallows, and flycatchers (identified remains include a single flycatcher species, *Tyrannus vociferans* (Cassin’s kingbird)). It is unclear whether they were not present near La Brea, they have simply not yet been identified among the currently unstudied remains, their remains have yet to be recovered, or they were present at the site but behaviorally unlikely to become entrapped. For example, perhaps aerial insectivores such as swifts or swallows avoided the tar. In addition, the list of passerines seems to include fewer migrant species than sedentary ones, and it is possible that migrants were in the area of La Brea too briefly during migration to become entrapped, at least in high enough frequency to have been detected to date. Changes in species diversity between the LGM and the present could be a result of changes in niche breadths or the number of niches for which the site presents suitable conditions at a given time, and testing these factors is possible through (climatic) niche modeling.

Here, we use the bird record of La Brea and ecological niche modeling to meet four objectives. First, we compare predicted LGM breeding and wintering distributions for landbird species (n = 86) identified (to species or genus) from La Brea to determine if niche models successfully predict species’ presence. This provides a check on the validity of niche models for predicting LGM distributions. We also estimate the degree of species turnover. Secondly, for 97 species not yet identified from La Brea but found within or near the region today, we create breeding and wintering season niche models to predict which species might have been at La Brea, thereby creating a prospective checklist of birds. Third, we tally changes in seasonal status (resident, breeder, migrant) to evaluate the stability of life histories over the 21 millennia represented by the avifauna at La Brea. Lastly, we determine whether a guild of bark-foraging birds showed quantitative shifts in Eltonian niche breadths between the present and the LGM.

## Methods

We constructed breeding season and wintering season ecological niche models for 63 landbird species documented from La Brea, representing considerable taxonomic diversity (Table 1) including 41 residents, 3 breeders, 11 winter visitors, 1 extinct, 1 introduced, and 6 that are not today found within 100 km of La Brea. In addition, we considered 23 species that are congeneric with taxa identified only to genus in the La Brea list. To determine if other species could have been present, we selected 97 additional species that are today found within or near the La Brea region but not among the identified remains (to species or genus), and determined whether niche models predicted their occurrence at the site at the LGM; these included 30 breeding, 2 migrants, 44 residents, 14 winter visitors, and 7 species nearby but not present within 100 km of the tar pits. We excluded species associated with water (waterfowl, shorebirds), which are not easily amenable to niche modeling. We consider the wild turkey as not present today owing to well-documented recent introductions. We count the existence of three extinct species of landbirds [12–14], one extinct owl (*Strix brea*; [15]), and the passenger pigeon (*Ectopistes migratorius*), which we include in the overall tally of species but exclude from niche modeling. We also excluded the northwestern crow (*Corvus caurinus*) because Johnston [16] has shown that it is not distinguishable phenotypically from the American crow (*C*. *brachyrhyncus*). Howard [11] listed the number of specimens and number of pits (out of 13) for each species; we note that at least nine species are represented by a single specimen, and 25 species by five or fewer specimens (Table 1). Howard (11) did not list the number of specimens of pine siskin (*Spinus pinus*) or cedar waxwing (*Bombycilla cedrorum*) but these species are in the online list. We used maps of bird distributions (https://www.birds.cornell.edu/) to determine species’ present status; when distribution maps were unclear, we considered a species present if three or more locations from the breeding bird survey were represented in the 100-km area surrounding La Brea.

We estimated the general LGM ranges of species using niche modeling and the 19 Bioclimatic variables [17]. Species modern localities were obtained from the breeding bird survey (https://www.pwrc.usgs.gov/bbs/; accessed multiple times); only localities west of -104° longitude were used to restrict analyses to areas likely most relevant to La Brea. We entered locality information into Maxent [18,19] to build a climatic niche model that was then projected onto the LGM climate layers using DIVA-GIS [20] (~20 ka; CCSM model); we used default parameters with the exception that we used 1000 iterations to assist model convergence. To explore the influence of default parameters, we reanalyzed 100 species at random (split between breeding and wintering) with 5000 iterations, and no clamping or extrapolation. We recognize that the specimens documented at La Brea might reflect entrapment of wintering and migrant species. To expand discovery of species occurrences at La Brea, we plotted potential winter distributions by downloading January occurrences from the Global Biodiversity Information Facility (https://www.gbif.org; Appendix 2) and built niche models for each species using the same 19 Bioclim layers. If there were areas in the range with a high density of points, we randomly sampled up to1500 breeding sites. We did not prune the climate layers for winter-only months [21–23] because we believe that for birds, the entire year is relevant to the existence of plant species at the site that in turn dictate avian presence. That is, if a plant species cannot survive the entire year, it will not be present at the site, nor will the birds that depend on it. Thus, for both breeding and wintering, we assume that all of the Bioclim layers are relevant. In addition, there is not a “summer” and “winter” seasonal period that is the same for all birds, especially for species that only migrate past La Brea. We note that very few studies delete winter months for estimating breeding distributions, in our opinion for the same reason. S1 and S2 Tables contain the breeding and wintering locality data, respectively, used in the models.

A myriad of different modifications have been proposed to tweak niche models [24–25]. Our goal in niche modeling was not to identify the exact range of a species at the LGM, instead we wished to estimate whether the 187 focal species were present at or within 100 km of La Brea. We used the 10% probability threshold to depict presence or absence at the LGM [26], and we recorded the distance from La Brea to the nearest predicted occurrence. We recognize there are multiple possible thresholds but in a comparison of a wide range of different threshold values for 50 species we found little change in our results. Some authors suggest using a correlation analysis to reduce the number of bioclimatic variables, by deleting one of two variables correlated at or above some level. We do not find this appropriate because any cutoff used is arbitrary. In addition, we analyzed species using the same bioclimatic variables; it is doubtful that all species would respond in the same way to a reduced set of variables (see below). For example, Zink and Gardner [27] analyzed multiple species using all 19 bioclim variables, and found that each variable contributed significant to at least one species, but if a correlation analysis had been used to eliminate variables, this explanatory information would have been lost. Hence, we kept all 19 variables in our analyses.

Nonetheless, to explore the possibility of bias in the above-described data sets and modelling approach, we made new niche models for 103 randomly chosen species using the MIROC−ESM_LGM climate layers. For this random sample of the species we thinned the locality data to only include records > 20 km apart for each species to account for spatial sampling bias using the package spThinn [28]. To explore the effect of the background area selection for the model, we selected the study area for modelling each species niche as the minimum convex polygon of locality records surrounded by a 150 km buffer. We compared the results of these models with the previously described ones to determine if systematic bias stemming from differences in niche construction methods influenced our results.

The area surrounding La Brea includes a range of elevations from near sea level (La Brea = ca. 60 m) to over 1500 m, supporting differing habitats altitudinally. For example, southeast of La Brea the elevation is similar for 65 km, ranging from sea level to 100 m. Elevations reach 1500 m within 40 to 75 km of La Brea to the northeast and northwest, although there are intervening areas less than 150 m. This elevational heterogeneity complicates scoring a species as present at La Brea from niche models. Given the mobility of most birds [29], one might assume that if a LGM distribution map predicted presence within 100 km of La Brea, the species was likely present there. However, as noted above, some environments within 100 km from La Brea are very different in both elevation and habitat. We plotted the distribution of distances from La Brea to the closest predicted occurrence for each species in breeding and winter periods, and we considered a distance of 100 km or less as indicating presence at La Brea. Although 100 km might seem a large distance for species to traverse non-optimal habitat, over seasons and thousands of years, we considered it a biologically reasonable threshold distance. If a species is within 100 km in both breeding and wintering seasons, we considered them resident. As a control, one can examine the niche models for eastern species and observe that they do not predict presence at La Brea [27].

To explore whether species’ niche breadths changed over time, we selected a guild of bark-foraging species including Nuttall’s woodpecker, hairy woodpecker, downy woodpecker, black-backed woodpecker, acorn woodpecker, red-shafted flicker, Williamson’s sapsucker, red-breasted sapsucker, white-headed woodpecker, pileated woodpecker, Lewis’ woodpecker, red-breated nuthatch, white-breasted nuthatch, and brown creeper. Niche breadth was estimated by applying the inverse concentration metric of Levins [30] as implemented in ENMTools [31–34], for both breeding and wintering periods at the LGM and present. To compare niche breadths we computed Pearson rank-order correlation coefficients to between scores from the two time periods to mitigate empirical differences. We computed Simpson’s [35] measure of species turnover as “min (b,c)/[a + min(b,c)] “, where b = number of species unique to La Brea (19), c = number of species unique to present (2), a = number of species present at both time intervals (187–21 = 166).

## Results

### Landbird species identified from La Brea during the LGM

**Fig 1:**
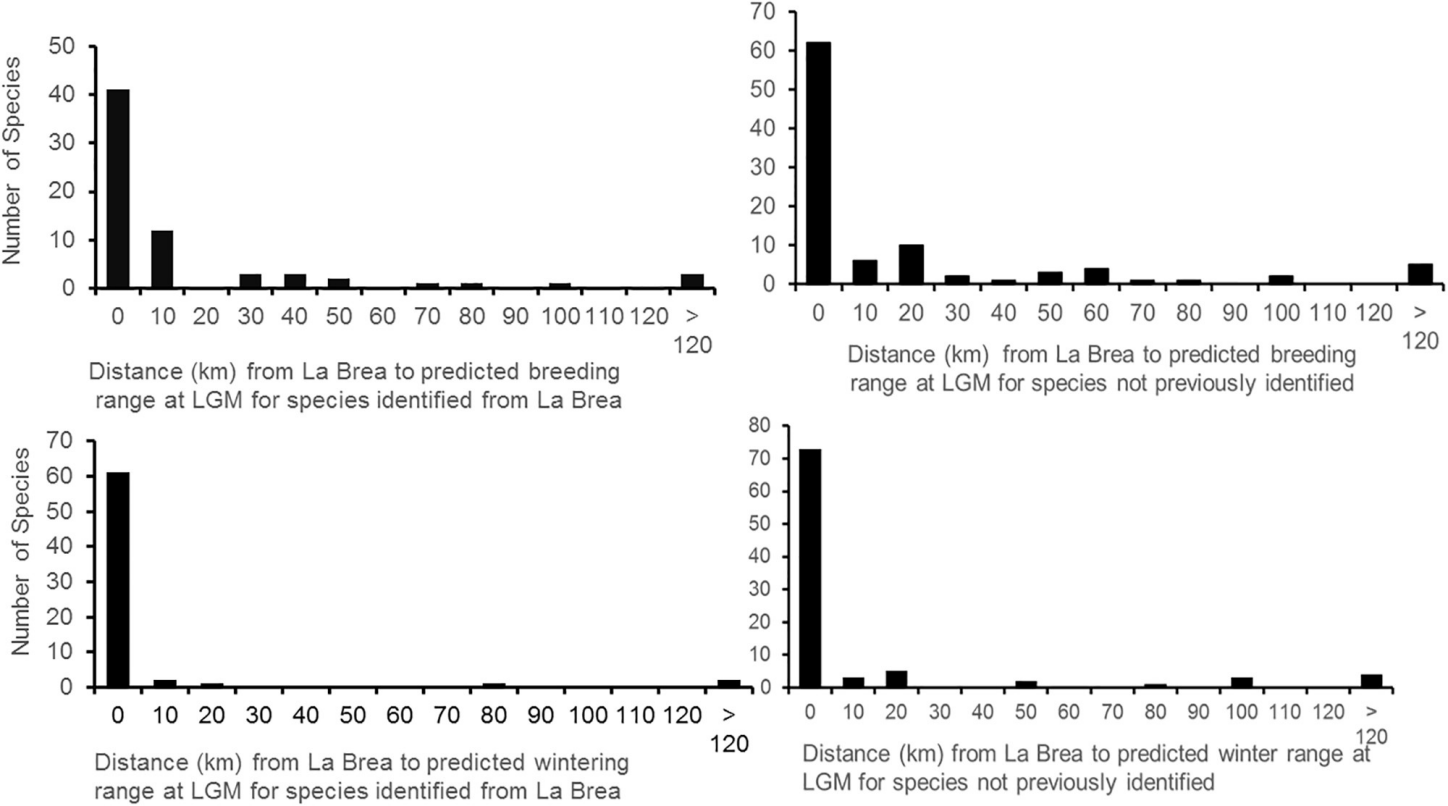
Distances from predicted distributions for birds documented or predicted to be breeding at La Brea.

For the 63 documented extant species we examined (S1 Table), niche models showed that 36 species (58%) had ranges that overlapped La Brea at the LGM, 49 species (78%) were within 20 km, and 60 (95%) species were within 100 km (Fig 1). Of the 63 species, five are not present today within 100km, suggesting range shifts, but less than 500 km. The LGM distribution of the Chihuahuan raven (*C*. *cryptoleucus*) was inconsistent with presence at La Brea but the possibility exists that the single specimen was misidentified. For specimens identified only to genus from La Brea, we evaluated congeneric species occurring locally at the present time (Table 1), finding that 22 of 23 species were predicted to have been within 100 km of La Brea at the LGM.

**Fig 2:**
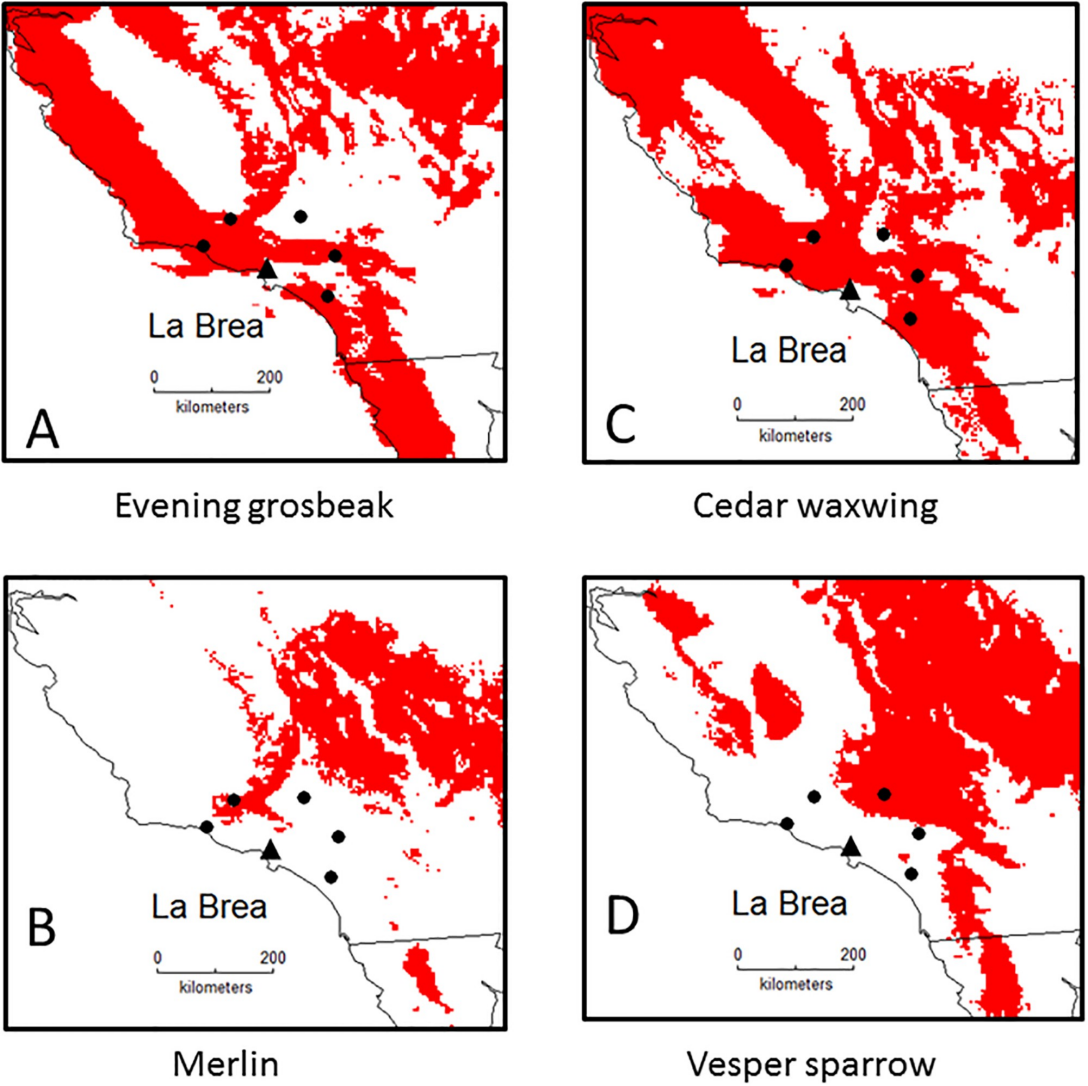
Predicted Last Glacial Maximum breeding distributions of four species. The triangle indicates the location of La Brea, and the five filled circles are 100 km from La Brea.

**Fig 3:**
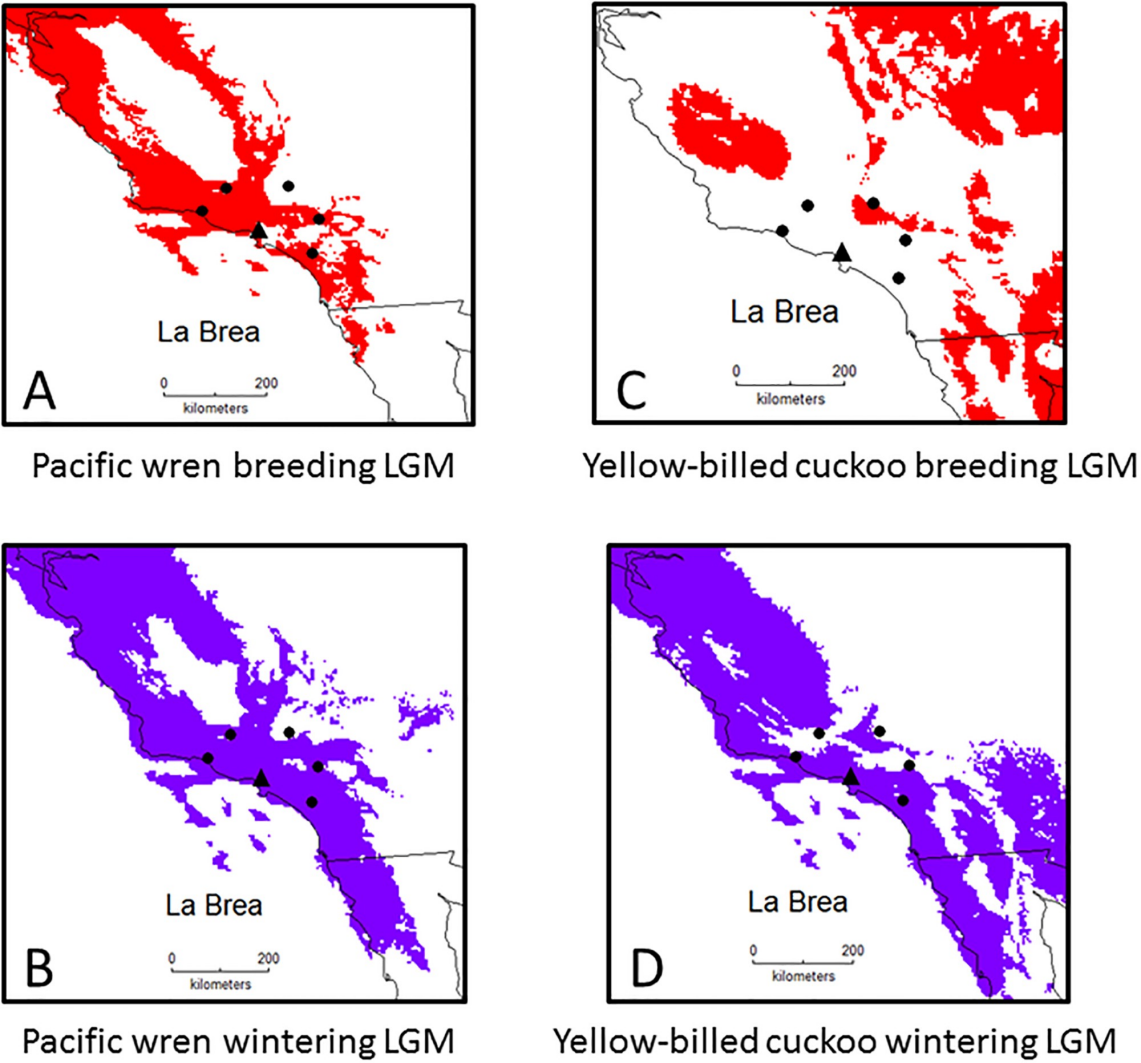
Predicted breeding and wintering distributions for two species suggesting resident status at the LGM.

For the 63 verified La Brea species that are extant, 41 (65%) species were predicted to have the same life history at the LGM and present, whereas 24 (35%) showed shifts, most involving shifts from resident status at the LGM, with the largest frequency being 11 residents that became winter visitants (Table 1). For example, in Fig 2 we show LGM breeding distributions for four species that currently only winter within 100 km of La Brea, but were breeding and wintering at the LGM, resulting in their shift to resident status. The Pacific wren and yellow-billed cuckoo are not present today near La Brea, whereas they were residents at the LGM (Fig 3). For the 23 species from genera identified from La Brea, a similar distribution of life history shifts was found, with 16 (70%) species being consistent across time, and seven species showing shifts (Table 2). Nineteen species that were present at the LGM are absent today (3 breeding, 1 wintering, 15 residents) and one species not present at the LGM is today a breeding species.

**Table 2:** Shifts in residency and/or migratory behavior in birds documented or potentially present at La Brea.

Status: Present—LGM	Documented Species	Species presence inferred from niche model	Congeneric Species	Totals
breeding—breeding	0	8	3	11
breeding—not present	1	2	0	3
breeding—resident	0	2	0	2
migrant—migrant	0	0	0	0
not present—breeding	0	0	1	1
not present—not present	1	1	0	2
not present—resident	0	1	0	1
resident—breeding	3	21	3	27
resident—extinct	5	0	0	5
resident—migrant	0	2	0	2
resident—not present	4	4	2	10
resident—resident	41	41	13	95
resident—winter	11	10	1	22
winter—breeding	0	1	0	1
winter—not present	1	0	0	1
winter—resident	0	2	0	2
winter—winter	0	2	0	2
Totals	67	97	23	187

### Landbird species not identified from La Brea

For the 97 species that have not yet been identified at La Brea, 95% were predicted to have occurred within 100 km during the breeding season and 91% in winter (Table 1). A total of 51 (54%) species were predicted to have the same life history at the LGM and present, whereas 38 (40%) showed shifts, most being shifts from resident status, with the largest frequency being 21 breeding species that became breeding-season only inhabitants (Table 1; excluding species that were not present at one or both times). Six species that were present at the LGM are absent today (2 breeding, 4 residents), and one species not present at the LGM is today a resident species. Across all categories, residents comprised 88% of the total species at the LGM and 60% at the present time.

### Niche breadths of bark-gleaners

**Fig 4:**
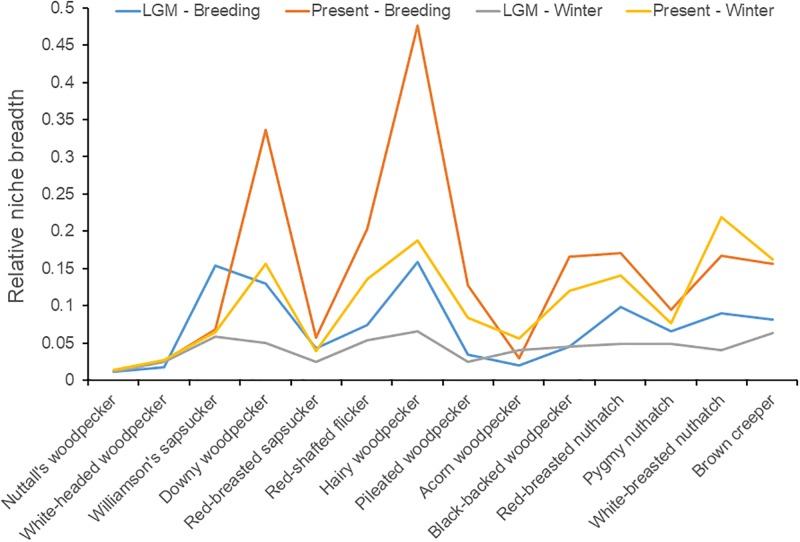
Comparisons of niche breadths in bark-gleaning birds at present and the LGM across seasons.

Our measure of niche breadth varied little between seasons and time periods (Fig 4), with the exception of downy woodpecker and hairy woodpecker, two of the more widespread woodpecker species. Overall, Pearson rank-order correlation coefficients were all > 0.7 and statistically significant (Table 3), suggesting no major shifts in niche breadth across time. Given 19 species unique to the La Brea record, and 2 unique to the present, and 166 species present at both time intervals, species turnover was low (Simpson’s [35] value = 2/168 = 0.012).

**Table 3:** Pearson product-moment correlation coefficients between measures of niche breadth for a guild of bark-foraging species.

	LGM breeding	Present breeding	Present winter
Present breeding	0.776**		
LGM winter	0.868**	0.723**	
Present winter	0.754**	0.890**	0.701*

** *P* < 0.01,

*P < 0.05

### Comparison of different niche modeling assumptions

**Fig 5:**
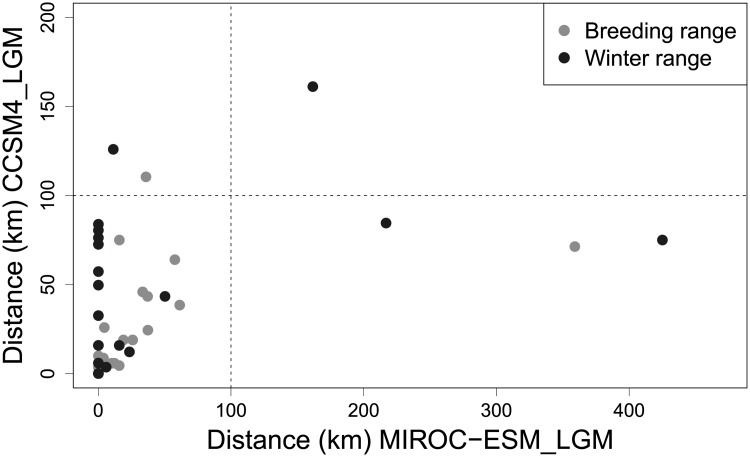
Plot of distances from La Brea to closest predicted occurrence under two different climatic conditions (CCSM4, MIROC-ESM) for 103 species of birds plotted as function of breeding or wintering ranges, showing only six species in which both analyses fail to predict occurrence within 100 km. The point at 150,160 is predicted by both analyses not to occur (verdin in winter) and hence is not in conflict. The two sets of predictions are significantly correlated (Pearson correlation = 0.50, *P* < 0.0001).

For the 103 species modeled under the MIROC−ESM_LGM conditions, we found that for five species (breeding season: yellow-billed cuckoo, warbling vireo; winter season: ash-throated flycatcher, yellow-billed magpie, Northwestern crow) our conclusions about presence or absence within 100 km of La Brea were altered (Fig 5). Therefore, the two different sets of niche modeling assumptions agreed on 95% of the species.

## Discussion

Many ecological principles were derived from lists of species of modern organisms from different continental areas or from different islands. Given our understanding of glacial history in north temperate regions, it is obvious that species ranges changed with the onset and retreat of glaciers. Specifically how each species responded is not clear because in essence we lack field guides to the past distribution of species. Niche modeling provides a way to construct species lists for communities at different time periods [4], such as the LGM. However, niche models are hypotheses and not based on direct observational information, as are modern checklists. In this study, nearly all of the species identified from skeletal remains at La Brea were predicted to have occurred there or within 100 km by ecological niche models (S1 Table, Fig 1). Although this comparison represents a sample at just one geographical site, it nonetheless lends confidence to the ability of niche reconstructions to produce reasonably accurate LGM range estimates, at least for birds.

The success of niche models in predicting species already known to occur at La Brea at the LGM makes it possible to predict which other species ought to have been present at the site. Of the 97 species currently unverified from La Brea, only two were predicted not to have occurred within 100 km of the site (Table 1). The lack of specimens of thrushes, hummingbirds, vireos, wrens, among others, suggests either that it was relatively rare for these birds to be trapped in the tar, their migratory habits resulted in short-term presence at La Brea, or they simply have not been identified from remains already or as yet to be recovered. A large proportion could be awaiting identification in the remains from La Brea. K. Campbell (email to RMZ on 3 June 2019) remarked “there are probably tens of thousands of passerine bones in the collection that have never seen the light of day". Our analysis (S1 Table) therefore provides a prospective checklist of land bird species at La Brea at the LGM, one of the first such checklists produced with the aid of niche modeling. Descriptions of species ranges at the LGM will facilitate comparison of changes in avifaunal composition over the last 21,000 years.

Of the 187 total species examined (including five extinct species), 183 were present at the LGM in one or more seasons, whereas 166 are present today. Thus, species richness decreased from the LGM to the present. In many studies of species turnover in birds (e.g., [1]), previous baseline surveys were judged inadequate. In the case of La Brea, we suggest that the species lists for both time periods are relatively robust, and there is relatively little turnover (Simpson’s [35] value = 0.012), and differences in species occurrence are due mostly to relatively local range shifts rather than species disappearance. On the other hand, the niche models (Table 2) commonly implied shifts in residency and life history status. Across all categories, and considering only species present at both time periods (169), 56 species (33%) shifted from one migratory state to another (Table 1), with the commonest being a larger number of resident species at the LGM (161; 88%) than at the present time (100; 60%). In particular, 27 species switched from resident at the LGM to being breeding-season only today, implying a suitable year around seasonal environment and the cessation of migration at the LGM (Table 2). These shifts resulted in greater species diversity in the breeding season (residents and breeding species), with 177 species estimated at the LGM and only 140 at the present. This suggests considerable plasticity in life history strategies, with frequent transitions from resident to migratory status [27].

Zink and Gardner [27] suggested that many current long-distance migrants reverted to being tropical sedentary residents during glacial maxima. However, most species that retained LGM breeding distributions in North America were in the western part of the continent. Peterson et al. [36] discovered that many niches do not change until well after speciation, which suggests niche conservatism over considerable periods. A greater percentage of resident species suggests a different niche structure than at present, such as narrower niches. However, we did not observe any strong trends in niche breadths in our sample of bark-foragers in any season or time period (Fig 4), although most were residents at both periods. This suggests that the LGM climate was suitable to a greater number of species, rather than changes in niche breadth that could allow greater species packing (e.g., niche partitioning). Warren (in litt.) suggested that niche breadth metrics are affected by the fact that environmentally suitable habitat for birds was more common or more uniformly distributed at the LGM. Thus, although niche conservatism may well be a characteristic of many birds [37], these niches can be seasonally variable. Future studies should consider a null model approach to account for the expected differences based on available habitat.

It is unclear what the vegetation at La Brea might have been at the times most of the specimens were deposited. Fragomeni and Prothero [38] wrote that study of offshore sea cores by Heusser [39] suggested that “the region changed from oak and chaparral vegetation around 59 ka to pine-juniper-cypress woodlands by 24 ka, then to a closed-cone juniper-ponderosa forest with abundant winter snow during the last glacial maximum (24–14 ka).” This could be inconsistent with our suggestion that there were more residents than migrants in the La Brea avifauna; however, if the dates given for the duration of this environment are actually older, there could be no inconsistency. Given changes in community vegetation structure, it is of interest that stasis in the size and shape of La Brea mammals has been noted [38,40].

Because of the many different assumptions used in published niche models [41–44], we explored the effects of LGM climate projections from different Global Climate models (CCSM, MIROC−ESM_LGM), as well as the effect of background (accessible) area selection, and spatial sampling bias. Our criterion was simply whether each model predicted occurrence within 100 km of La Brea, and we found that 95% of the models led to the same conclusions, showing our results are robust to varying climatic data and niche modelling approach. There is, however, clearly differences in the projected distributions at scales less than 100 km (Fig 5), which could be further explored for answering different questions. Nonetheless, it appears that these differences stem mainly from differences in the Global Climate Models.

## Supporting information

S1 Table Locations (longitude, latitude) for each specimen used in breeding niche models. (XLSM)

S2 Table Locations (longitude, latitude) for each specimen used in wintering niche models, including information on downloads from Global Biodiversity Information Facility. (XLSM)
